# Neighbourhood socioeconomic status and cardiovascular risk factors: a multilevel analysis of nine cities in the Czech Republic and Germany

**DOI:** 10.1186/1471-2458-7-255

**Published:** 2007-09-21

**Authors:** Nico Dragano, Martin Bobak, Natalia Wege, Anne Peasey, Pablo E Verde, Ruzena Kubinova, Simone Weyers, Susanne Moebus, Stefan Möhlenkamp, Andreas Stang, Raimund Erbel, Karl-Heinz Jöckel, Johannes Siegrist, Hynek Pikhart

**Affiliations:** 1 Department of Medical Sociology, Heinrich-Heine-University Düsseldorf, Germany; 2 International Institute for Health and Society, Department of Epidemiology and Public Health, University College London, UK; 3 Centre for Environmental Health, National Institute of Public Health, Prague, Czech Republic; 4 Institute for Medical Informatics, Biometry and Epidemiology, University Duisburg-Essen, Germany; 5 Clinic of Cardiology, West-German Heart Center Essen, University Duisburg-Essen, Germany; 6 Institute of Medical Epidemiology, Biometry and Informatics, University Hospital of Halle, Germany

## Abstract

**Background:**

Previous studies have shown that deprived neighbourhoods have higher cardiovascular mortality and morbidity rates. Inequalities in the distribution of behaviour related risk factors are one possible explanation for this trend. In our study, we examined the association between cardiovascular risk factors and neighbourhood characteristics. To assess the consistency of associations the design is cross-national with data from nine industrial towns from the Czech Republic and Germany.

**Methods:**

We combined datasets from two population based studies, one in Germany ('Heinz Nixdorf Recall (HNR) Study'), and one in the Czech Republic ('Health, Alcohol and Psychosocial Factors in Eastern Europe (HAPIEE) Study'). Participation rates were 56% in the HNR and 55% in the HAPIEE study. The subsample for this particular analysis consists of 11,554 men and women from nine German and Czech towns. Census based information on social characteristics of 326 neighbourhoods were collected from local administrative authorities. We used unemployment rate and overcrowding as area-level markers of socioeconomic status (SES). The cardiovascular risk factors obesity, hypertension, smoking and physical inactivity were used as response variables. Regression models were complemented by individual-level social status (education) and relevant covariates.

**Results:**

Smoking, obesity and low physical activity were more common in deprived neighbourhoods in Germany, even when personal characteristics including individual education were controlled for. For hypertension associations were weak. In the Czech Republic associations were observed for smoking and physical inactivity, but not for obesity and hypertension when individual-level covariates were adjusted for. The strongest association was found for smoking in both countries: in the fully adjusted model the odds ratio for 'high unemployment rate' was 1.30 [95% CI 1.02–1.66] in the Czech Republic and 1.60 [95% CI 1.29–1.98] in Germany.

**Conclusion:**

In this comparative study, the effects of neighbourhood deprivation varied by country and risk factor; the strongest and most consistent effects were found for smoking. Results indicate that area level SES is associated with health related lifestyles, which might be a possible pathway linking social status and cardiovascular disease. Individual-level education had a considerable influence on the association between neighbourhood characteristics and risk factors.

## Background

Socio-economic inequalities can be analysed at different levels, from the individual to urban neighbourhoods up to cities and whole countries. The relationship between individual social characteristics and health is well documented [1]. But even at the aggregate level socioeconomic status (SES) seems to be associated with health, as a growing number of studies on relations between neighbourhood-level SES, mortality and morbidity demonstrate [2]. For instance, several studies found a higher all-cause mortality in deprived urban neighbourhoods compared to areas with higher social status [3-7]. This relationship is especially distinct for mortality due to cardiovascular causes [8-11]. Moreover, indicators of morbidity are also unequally distributed by neighbourhood SES, for example non fatal coronary heart disease [12-15] or a self rated poor health [16,17].

These effects are usually statistically controlled for individual-level socioeconomic status and in general the effect of neighbourhood SES persists after adjustment for personal social characteristics [18], indicating an independent influence of area deprivation on health.

A number of intermediate factors linking neighbourhood SES to individual health have been proposed. Among such factors, there are crime rate, pollution, noise, social stress and a lack of health related facilities and structures. The promotion of unhealthy lifestyles in an adverse socioeconomic environment is another possible pathway. Such an association is documented for smoking, where rates are significantly higher in low SES neighbourhoods, irrespective of personal characteristics [16,19-22]. Other risk factors are less frequently analysed, but there is emerging evidence for an association, e.g. for overweight, low physical activity and hypertension [23-27].

In this project, we examine the evidence for area-level inequalities of health damaging lifestyles by using a set of cardiovascular risk factors in multilevel-analysis. Area level social inequalities in the distribution of behaviour related individual risk factors are of particular interest in this field of research, because they address a pathway by which the broader social environment could influence the health of urban populations. The design of the study is cross-national, since we analyse data from a German and a Czech study with urban populations from nine cities. Comparative analysis allows evaluation of the strength of associations between neighbourhood SES and risk factors in countries that are at different stages of their economic development [28]. The Czech Republic is a former communist country and currently in a fundamental transition to a market-oriented capitalist society. But irrespective of an impressive improvement in living conditions, the current process of transition is still characterized by threats to the well-being of large population groups who are exposed to material deprivation and social instability [29,30]. No contextual study has been conducted in this particular region before, and to our knowledge only one study exists in which area-level social inequalities in an Eastern Europe country (Moscow, Russia) after the breakdown of communism have been investigated [31].

Germany, on the other hand, is a highly developed country and charter member of the European Union, but has been faced with economic decline in the years under study, especially in the urban region where the study has been conducted. A cross-national and cross-town comparison of the results gives an idea about the consistency of associations between neighbourhood-level SES and cardiovascular risk factors. Such a comparison is useful, because the countries represent different stages of the development of modern industrialized countries. The importance of the characteristics of the place of living for individual health might vary in relation to the larger social, cultural and economic context. The present paper explores the extend to which such a variation exists.

## Methods

We report a comparative analysis of data from the baseline screenings of two longitudinal cohort studies. Both studies examined men and women from urban populations, one in Germany (the 'Heinz Nixdorf Recall (HNR) Study'), and one in the Czech Republic (the 'Health, Alcohol and Psychosocial Factors in Eastern Europe (HAPIEE) Study'). The sample of the HNR study was randomly selected from population registers of three neighbouring Western German industrial towns Bochum, Essen, and Mülheim. Baseline examination took place during the years 2000 to 2003. The Czech sample was also drawn randomly and was based on registers from the six cities Havirov/Karvina, Hradec, Jihlava, Kromeriz, Liberec and Usti nad Labem Baseline investigations started in 2002 and ended in 2005.

Participation rate was 56% in Germany, giving an overall sample size of 4,814, and 55% in the Czech Republic with a resulting sample size of 8,856 participants. Sampling methods and recruitment are described in detail by Stang and colleagues [32] for the HNR study and by Peasey and colleagues [33] for the HAPIEE study. To ensure comparability of the two studies we restricted our samples to participants aged 45 to 69 years at baseline, the overlapping age range between the studies. Furthermore, all participants who reported a history of myocardial infarction were excluded (171 in HNR; 448 in HAPIEE). Lastly, in the HAPIEE study it was impossible to merge small-area data for 645 participants (missing area information or incomplete addresses). Altogether the resulting effective sample size for this analysis was n = 4,032 for the HNR study and n = 7,522 for the HAPIEE study.

In the HNR study, computer-assisted personal interviews were conducted and supplemented by paper and pencil questionnaires [34]. Special efforts were invested in quality control of data collection and data handling, as documented in an external certification (DIN EN ISO 9001). In the HAPIEE study, data were collected by paper and pencil questionnaires [[33], for details see [35]]. In addition, physical examinations were conducted in both studies by trained study personnel which assessed blood pressure, body weight and height and many other parameters. Only variables measured in a comparable way in both studies were included.

### Individual level cardiovascular risk factors

Four established cardiovascular risk factors represent the risk profile of the participants: (1) obesity, (2) hypertension, (3) current smoking and (4) low physical activity. Obesity is defined by a body-mass-index ≥ 30 kg/m^2^. Blood pressure was measured with an oscillometric device (HNR=Omron HEM-705-CP; HAPIEE=Omron M5-I) and mean values of the 2^nd ^and 3^rd ^measurement taken at least three minutes apart were calculated [36]. We classified participants as hypertensive if they had a systolic value ≥ 160 mmHg or a diastolic value ≥ 90 mmHg or were taking regular antihypertensive medication. Smoking habits and leisure time physical activities were both assessed by standardized questionnaires. Current smokers and participants with low physical activity (frequency of regular leisure time physical activity/sports less than once a week) were defined as being at risk.

### Individual-level socioeconomic status

Socioeconomic status of the participants was operationalised by their educational degrees. The respective variable was classified according to the International Standard Classification of Education [37]. This classification indicates the highest educational degree by combining school and vocational training. Four categories were defined: primary, vocational, secondary and university degree.

### Individual-level covariates

Age and sex were adjusted for in all multivariate analyses. Additionally, economic activity and social isolation were included as covariates. All four variables could be considered as possible mediating or confounding factors in the association between neighbourhood-level SES and health. Economic activity is relevant, because it includes individual unemployment. The variable distinguishes four groups: participants still working, retired persons, unemployed and economically inactive people (i.e. housewife). Social isolation is a strong determinant of health [38] and is influenced by the socioeconomic context in which a person lives [39]. As the questionnaires about social network were not fully identical we used a simple operationalisation: people who reported to have regular contact with friends or relatives less than once a month were defined as socially isolated.

### Neighbourhood-level socioeconomic status

To measure neighbourhood SES, participants were linked to their area of residence using the baseline home address. Area of residence was defined by existing administrative boundaries in both countries. In the Czech Republic participants were nested in 220 neighbourhoods with a median number of 3517 inhabitants; in Germany a total of 106 geographical units were linked, with a median size of 11,263 inhabitants. In the next step, the area code was used to merge the neighbourhood data. Two indicators of neighbourhood level SES were available for this analysis: unemployment rate and overcrowding. Both indicators are frequently used in area-level research, e.g. as part of the Townsend or the Carstairs deprivation indexes [40]. Unemployment is a strong indicator for material deprivation [22] in a neighbourhood, while overcrowding reflects aspects of social stress and physical hazards due to a high population density [41,42]. Respective information was obtained from the local census authorities of the towns. The unemployment rate (%) was calculated by dividing the number of unemployed in the area by the number of the economically active population (unemployed + working population). In both studies an indicator of overcrowding was calculated by dividing the total living space (houses/flats) in m^2 ^by the total number of inhabitants. To ensure comparability between the countries we examined relative instead of absolute differences in neighbourhood SES. Analogous to a recent comparative study of van Lenthe and colleagues [28] the continuous variables were therefore grouped into four categories by calculating country-specific quartiles. The variables were coded from 1 to 4, where group 1 was the reference group with the highest SES having an unemployment rate in the lowest quartile or a mean number of m^2 ^per inhabitant in the highest quartile.

### Statistical analysis

We used bivariate statistics (proportions/chi^2^-test; means/t-test) to summarize the characteristics of the samples and the distribution of the socioeconomic status indicators. Due to the hierarchical structure of the data it was necessary to apply multi-level statistical methods for the analyses of neighbourhood- and individual-level effects. The two country-datasets were analysed separately, and differences are interpreted qualitatively on the basis of the estimates derived by the multi-level regression models. The four risk factors were defined as the dependent variables in the regression models. A logistic regression model with mixed effects was used for statistical analysis. For the fixed effects the following two sets of variables were used: a) individual characteristics: age, gender, socioeconomic status, isolation and economic activity; b) contextual characteristics: unemployment and overcrowding.

For the random effects we analysed two types of nested structure:

1) random variability between cities and between districts within cities

2) fixed effects for cities and random effects for districts.

We decided between these possible model structures by evaluating the estimated variance components. If these model parameters were estimated as zero we reduced the model to a simple model without random effects.

The model parameters were estimated by the Penalized Quasi Likelihood method [43]. 95% confidence intervals are reported for odds ratios. The analysis was performed with fixed effects variables entered sequentially. Missing values were excluded. In general the item nonresponse was low, with the exception of obesity measures in the Czech Republic, where more than 1000 study participants had missing data.

For each outcome, the analysis was done in three steps. The first model contained only the area-level indicator and age, sex as covariates. In the second model, individual-level education was additionally included, and finally economic activity and social isolation were added in the third model. Analyses revealed only small differences of results between men and women. Therefore, numbers are given for the total group.

Descriptive statistics were calculated with the SPSS statistical package 12.0.1 and multilevel regression models with R version 2.3.1 (R Development Core Team 2006).

## Results

The distribution of the study variables in the German and the Czech sample are described in table 1. With the exception of age, all characteristics vary significantly between the two datasets. Due to different educational systems vocational degrees are more common in Germany compared to the Czech Republic with more secondary educational degrees. Furthermore, in Germany the unemployment rate is higher than in the Czech Republic, but social isolation was more common among Czechs. The risk factor prevalence was higher for three variables in the Czech Republic (obesity, hypertension, smoking), but the rate of physically inactive people was higher in Germany. The two neighbourhood-level indicators differ in their distribution too. The unemployment rate was higher in Germany, but the mean living space per person was nearly twice as high in Germany than in the Czech Republic. As mentioned above, the area-level variables were grouped according to country specific quartiles of the distribution for the multi-level analyses.

**Table 1:** Characteristics of the study samples

	**Czech Republic**	**Germany**	p*
*Participants, n*	7522	4032	
			
**Individual level variables**			
			
*Mean age *(± *SD*)	57.8 (± 6.9)	57.7 (± 6.6)	0.281
			
*Women, % (number)*	55.2 (4150)	51.5 (2075)	<0.001
			
*Education, % (number)*			
primary	12.5 (934)	10.0 (401)	<0.001
vocational	36.9 (2761)	55.5 (2230)	
secondary	37.0 (2769)	16.4 (659)	
university degree	13.7 (1022)	18.2 (730)	
			
*Social isolation, % (number)*	5.5 (406)	3.0 (119)	<0.001
			
*Economic activity, % (number)*			
still employed	53.8 (4009)	46.2 (1859)	<0.001
retired	42.5 (3162)	32.3 (1299)	
unemployed	3.1 (228)	7.0 (282)	
inactive, other	0.6 (46)	14.4 (580)	
			
*Obesity, % (number)*	30.0 (1868)	26.4 (1059)	<0.001
			
*Hypertension, % (number)*	55.6 (4177)	41.7 (1680)	<0.001
			
*Smoking, % (number)*	26.9 (1996)	25.0 (1006)	0.028
			
*Low physical activity, % (number)*	30.8 (2230)	44.7 (1799)	<0.001
			
**Area level variables**			
			
*Unemployment rate, mean (SD)*	10.4 (± 5.6)	12.3 (± 3.4)	<0.001
			
*Overcrowding – m*^2^*of living space per person, mean (SD)*	18.8 (± 3.1)	38.9 (± 4.6)	<0.001

* chi-square test for categorical and t-test for continuous variables

The prevalence of the risk factors by country and level of neighbourhood deprivation is shown in table 2. With the exception of hypertension in Germany, the prevalence increases with an increasing unemployment rate in both countries. Some of the differences are considerably high, especially in the German dataset. The second part of the table demonstrates, that the trend is generally weaker for the overcrowding indicator.

**Table 2:** Proportions of participants with risk factors and with low education by quartiles of neighbourhood unemployment and overcrowding in the two countries

	**Quartiles of neighbourhood unemployment**				
		
	**1 (lowest unemployment)**	**2.**	**3.**	**4. (highest unemployment**)	p (chi^2^)
**% obesity**					
Czech Republic	29.2	27.2	31.2	32.2	0.010
Germany	20.9	27.2	28.1	29.6	<0.001
					
**% hypertension**					
Czech Republic	51.5	54.5	57.2	59.6	<0.001
Germany	39.7	42.1	41.7	43.4	0.392
					
**% current smoking**					
Czech Republic	26.0	23.1	26.9	31.8	<0.001
Germany	20.0	25.0	24.4	30.8	<0.001
					
**% low physical activity**					
Czech Republic	28.9	28.1	32.2	34.2	<0.001
Germany	39.5	43.7	46.4	49.1	<0.001
					
**% lowest education**					
Czech Republic	9.2	9.9	11.5	19.5	<0.001
Germany	6.2	7.6	11.1	15.1	<0.001

	**Quartiles of neighbourhood overcrowding**				
		
	**1 (no overcrowding)**	**2.**	**3.**	**4. (overcrowding)**	p (chi^2^)

**% obesity**					
Czech Republic	30.5	28.1	31.0	30.3	0.307
Germany	22.3	25.5	29.2	28.8	<0.001
					
**% hypertension**					
Czech Republic	55.1	56.0	55.2	56.3	0.875
Germany	40.3	42.2	42.2	42.2	0.772
					
**% current smoking**					
Czech Republic	23.8	25.2	26.7	31.8	<0.001
Germany	20.2	24.8	27.2	28.0	<0.001
					
**% low physical activity**					
Czech Republic	32.5	28.0	29.7	32.8	0.004
Germany	40.3	44.0	46.8	47.7	0.002
					
**% lowest education**					
Czech Republic	11.0	10.9	10.8	17.2	<0.001
Germany	6.8	7.1	11.4	14.7	<0.001

To what extent the bivariate associations are confounded by covariates was assessed in the multivariate hierarchical regression analyses. Main results for unemployment are presented in table 3. In the first age and sex adjusted model neighbourhood unemployment shows a small but consistent association with obesity, smoking and low physical activity in both countries. Odds ratios are higher in Germany than in the Czech Republic, a difference which persists even after further adjustment for personal education and other covariates.

**Table 3:** Risk factors in relation to the neighbourhood unemployment rate (quartiles) in Germany and Czech (odds ratios and 95%CI; multivariate, multi-level regression models)

**Risk factor **(number of observations included)	**Model 1 **adjusted for age, sex	**Model 2 **adjusted for age, sex, education	**Model 3 **adjusted for age, sex, education, economic activity, social isolation
	**Country = Czech Republic**		
**Obesity **(6082)			
1. lowest unemployment	1.00	1.00	1.00
2.	0.91 [0.80–1.05]	0.92 [0.82–1.03]	0.89 [0.78–1.02]
3.	1.16 [1.01–1.34]	1.09 [0.98–1.25]	1.11 [0.97–1.27]
4. highest unemployment	1.23 [1.04–1.46]	1.07 [0.91–1.26]	1.03 [0.87–1.21]
			
**Hypertension **(7316)			
1. lowest unemployment	1.00	1.00	1.00
2.	1.01 [0.88–1.52]	1.02 [0.90–1.16]	1.02 [0.90–1.17]
3.	1.03 [0.87–1.21]	1.03 [0.87–1.21]	1.06 [0.90–1.25]
4. highest unemployment	0.90 [0.69–1.18]	0.87 [0.67–1.13]	0.89 [0.69–1.16]
			
**Smoking **(7235)			
1. lowest unemployment	1.00	1.00	1.00
2.	0.90 [0.78–1.05]	0.90 [0.78–1.04]	0.92 [0.79–1.07]
3.	1.22 [1.02–1.46]	1.21 [1.01–1.43]	1.19 [1.00–1.43]
4. highest unemployment	1.46 [1.14–1.87]	1.32 [1.04–1.69]	1.30 [1.02–1.66]
			
**Low phys. activity **(7078)			
1. lowest unemployment	1.00	1.00	1.00
2.	0.98 [0.84–1.15]	0.97 [0.84–1.14]	0.97 [0.84–1.14]
3.	1.30 [1.09–1.54]	1.20 [1.01–1.42]	1.22 [1.03–1.44]
4. highest unemployment	1.36 [1.13–1.64]	1.11 [0.91–1.36]	1.12 [0.92–1.36]

	**Country = Germany**		
**Obesity **(3967)			
1. lowest unemployment	1.00	1.00	1.00
2.	1.41 [1.20–1.65]	1.35 [1.15–1.60]	1.34 [1.09–1.65]
3.	1.48 [1.24–1.75]	1.41 [1.17–1.68]	1.42 [1.20–1.69]
4. highest unemployment	1.60 [1.31–1.97]	1.48 [1.20–1.82]	1.50 [1.22–1.85]
			
**Hypertension **(3986)			
1. lowest unemployment	1.00	1.00	1.00
2.	1.09 [0.93–1.26]	1.08 [0.92–1.25]	1.06 [0.91–1.23]
3.	1.08 [0.93–1.26]	1.06 [0.90–1.24]	1.07 [0.91–1.25]
4. highest unemployment	1.18 [0.99–1.41]	1.14 [0.95–1.38]	1.14 [0.95–1.37]
			
**Smoking **(3986)			
1. lowest unemployment	1.00	1.00	1.00
2.	1.38 [1.16–1.63]	1.32 [1.11–1.56]	1.29 [1.09–1.53]
3.	1.32 [1.10–1.58]	1.25 [1.04–1.50]	1.24 [1.03–1.49]
4. highest unemployment	1.82 [1.47–2.24]	1.63 [1.32–2.02]	1.60 [1.29–1.98]
			
**Low phys. activity **(3981)			
1. lowest unemployment	1.00	1.00	1.00
2.	1.20 [1.00–1.46]	1.08 [0.93–1.35]	1.12 [0.93–1.35]
3.	1.36 [1.13–1.63]	1.22 [1.01–1.46]	1.22 [1.01–1.47]
4. highest unemployment	1.53 [1.23–1.90]	1.27 [1.02–1.57]	1.25 [1.01–1.56]

Adjustment for education (model 2) weakened the statistical association between unemployment and risk factors. In the Czech Republic odds ratios remained significantly elevated for smoking and low physical activity only. The introduction of the additional individual level covariates did not have an effect on the main estimates. Results are different in Germany where even after adjustment for education, significant estimates are observed for obesity, smoking and low activity. In both countries the association between neighbourhood unemployment and the dependent variables were particularly pronounced for smoking. With one exception the results are comparable for men and women (results for gender specific analysis not shown). This exception is obesity in Germany where the relationship with area level unemployment was more pronounced in women than in men. In example was the fully adjusted odds ratio for the highest compared to the lowest area unemployment 1.25 [0.92–1.69] in men and 1.74 [1.26–2.46] in women.

With regard to overcrowding, the associations are generally weaker than for unemployment (table 4). In the Czech Republic, the only significantly elevated odds ratios in the fully adjusted regression model were found for smoking. In Germany, the results for overcrowding and unemployment were similar in the first model. Adjustment for personal education reduced all area level estimates.

**Table 4:** Risk factors in relation to the neighbourhood overcrowding (quartiles) in Germany and Czech (odds ratios and 95%CI; multivariate, multi-level regression models)

**Risk factor **(number of observations included)	**Model 1 **adjusted for age, sex	**Model 2 **adjusted for age, sex, education	**Model 3 **adjusted for age, sex, education, economic activity, social isolation
	**Country = Czech Republic**		
**Obesity **(6082)			
1. no overcrowding	1.00	1.00	1.00
2.	0.84 [0.73–0.96]	0.83 [0.74–0.96]	0.85 [0.75–0.97]
3.	1.00 [0.86–1.16]	1.02 [0.88–1.16]	1.03 [0.90–1.19]
4. overcrowding	1.02 [0.85–1.22]	0.97 [0.81–1.13]	0.95 [0.80–1.12]
			
**Hypertension **(7316)			
1. no overcrowding	1.00	1.00	1.00
2.	1.10 [0.97–1.25]	1.11 [0.97–1.25]	1.09 [0.96–1.24]
3.	1.10 [0.95–1.26]	1.11 [0.96–1.27]	1.10 [0.96–1.27]
4. overcrowding	1.06 [0.89–1.25]	1.06 [0.89–1.26]	1.04 [0.88–1.24]
			
**Smoking **(7235)			
1. no overcrowding	1.00	1.00	1.00
2.	1.14 [0.97–1.33]	1.15 [0.99–1.34]	1.16 [0.99–1.35]
3.	1.18 [1.00–1.40]	1.20 [1.02–1.42]	1.19 [1.01–1.41]
4. overcrowding	1.33 [1.10–1.61]	1.28 [1.06–1.54]	1.28 [1.06–1.55]
			
**Low phys. activity **(7078)			
1. no overcrowding	1.00	1.00	1.00
2.	0.83 [0.71–0.98]	0.84 [0.72–0.97]	0.84 [0.71–0.98]
3.	0.90 [0.75–1.07]	0.90 [0.77–1.07]	0.91 [0.77–1.08]
4. overcrowding	1.01 [0.83–1.23]	0.94 [0.78–1.13]	0.93 [0.78–1.12]

	**Country = Germany**		
**Obesity **(3967)			
1. no overcrowding	1.00	1.00	1.00
2.	1.20 [1.01–1.42]	1.16 [0.98–1.37]	1.16 [0.98–1.36]
3.	1.47 [1.24–1.74]	1.40 [1.18–1.66]	1.40 [1.18–1.66]
4. overcrowding	1.43 [1.17–1.75]	1.32 [1.08–1.62]	1.33 [1.08–1.63]
			
**Hypertension **(3986)			
1. no overcrowding	1.00	1.00	1.00
2.	1.09 [0.94–1.26]	1.08 [0.93–1.25]	1.06 [0.91–1.23]
3.	1.12 [0.96–1.31]	1.10 [0.94–1.29]	1.09 [0.93–1.27]
4. overcrowding	1.11 [0.92–1.32]	1.07 [0.89–1.29]	1.06 [0.88–1.27]
			
**Smoking **(3986)			
1. no overcrowding	1.00	1.00	1.00
2.	1.32 [1.10–1.58]	1.28 [1.08–1.52]	1.28 [1.08–1.52]
3.	1.43 [1.18–1.72]	1.34 [1.12–1.60]	1.32 [1.10–1.57]
4. overcrowding	1.50 [1.19–1.88]	1.37 [1.09–1.71]	1.36 [1.09–1.69]
			
**Low phys. activity **(3981)			
1. no overcrowding	1.00	1.00	1.00
2.	1.18 [0.98–1.42]	1.12 [0.93–1.34]	1.13 [0.94–1.33]
3.	1.36 [1.12–1.66]	1.22 [1.01–1.47]	1.20 [0.99–1.45]
4. overcrowding	1.38 [1.12–1.73]	1.18 [0.95–1.46]	1.18 [0.95–1.47]

Concerning the fit of the multilevel models and the geographical variability between cities and districts, Germany and Czech showed different patterns (results not shown). For Germany, neither the city effect (random or fixed) nor the between district variability contributed substantially to the model after area-level SES indicators were introduced in the model. For Czech Republic the estimated components of variances suggested that there was more between area variability in the data than in Germany and random and fixed effects continue to contribute to the model fit even after an adjustment for the area-level variables.

In previous tables, education had a strong effect on the relationship between neighbourhood characteristics and risk factors. This is consistent with the strong associations between individual-level education and risk factors (table 5). In both countries an unhealthy risk factor profile was more common in participants with lower education. The high odds ratios indicate that individual level SES has a stronger impact on health risks than area level SES.

**Table 5:** Individual level socioeconomic status and outcome measures (odds ratios and 95%CI; multivariate, multi-level regression models, estimators adjusted for age, sex, education, economic activity, social isolation, neighbourhood unemployment)

	**Obesity**	**Hypertension**	**Smoking**	**Low physical activity**
	***Country = Czech Republic***			
University	1.00	1.00	1.00	1.00
Secondary	1.38 [1.22–1.55]	1.26 [1.14–1.39]	1.46 [1.31–1.64]	1.31 [1.17–1.46]
Vocational	1.98 [1.71–2.29]	1.52 [1.34–1.72]	1.91 [1.65–2.18]	2.44 [2.14–2.79]
Primary	2.58 [2.05–3.24]	1.24 [1.02–1.50]	2.22 [1.78–2.78]	3.69 [2.98–4.50]

	***Country = Germany***			
University	1.00	1.00	1.00	1.00
Secondary	1.32 [1.06–1.58]	1.20 [1.00–1.42]	1.42 [1.15–1.74]	1.38 [1.16–1.65]
Vocational	1.50 [1.27–1.78]	1.11 [0.96–1.29]	1.96 [1.64–2.34]	2.18 [1.87–2.53]
Primary	1.64 [1.23–2.21]	1.33 [1.02–1.73]	2.15 [1.57–2.95]	3.69 [2.81–4.84]

## Discussion

In this comparative study, we examined associations between two indicators of neighbourhood socioeconomic status and four cardiovascular risk factors. We made three main observations. First, in both countries, neighbourhood characteristics were related to the risk factors, but results differ by type of risk factor. Second, the shapes of the associations between socioeconomic status and the outcomes vary by country. Third, individual-level education appeared to play an important role in the relationships between neighbourhood characteristics and risk factors.

### Variation by type of risk factor

It is remarkable that in both countries a consistent contextual effect for the two area level indicators was evident for smoking. Individual level education had almost no effect on this relationship. This result is in line with previous studies [16,19-22,27,44]. Smoking behaviour seems to be consistently associated with the socioeconomic context in which people live. Several mechanisms explaining this association are currently debated. Van Lenthe and Mackenbach [45] hypothesize that neighbourhood stressors such as low security or noise pollution may be a possible link. They tested this explanation in a large Dutch cohort and found that adjustment for neighbourhood stressors substantially reduced the statistical association between neighbourhood disadvantage and smoking. Another hint is given by Chuang and colleagues [44]. The researchers analysed data from studies in northern California and found that the availability of tobacco, measured by the density and distance of convenience stores in the area, is an important determinant for smoking. Social norms and cultural beliefs define a further process by which socioeconomic context influences personal habits. A study by Curry and colleagues found evidence for this pathway [46]. In their examination, attitudes towards tobacco use were socially patterned by area-level characteristics. Such associations can be interpreted in a broader conceptual framework of cultural and social capital of communities [47,48].

Concerning the other three risk factors our study replicate earlier findings only partly. Independent neighbourhood-level effects for obesity and low physical activity were observed in Germany only, but for hypertension odds ratios were near one in both countries. This contrasts with studies where associations for all three indicators were found [23,25-27,49]. A possible explanation is the high prevalence of the risk factors in our sample. It can be concluded that some adverse lifestyles are rather common in the whole population, a fact that may reduce the strength of the effects exerted by the immediate socioeconomic environment on personal habits.

### Variation by country

Our study offers the opportunity to compare results from two countries with a different political, economic and societal structure. It is interesting to note that the relationship between neighbourhood socioeconomic status and risk factors was more pronounced in Germany. The reason might be that the overall standard of the infrastructure was lower in the Czech Republic for all neighbourhoods (and generally it was difficult to move from one area to another because a housing market was almost non-existent), so that the relative differences in respect to health-related characteristics of an area are small. To the contrary, in Germany standards of infrastructure are high, but relative inequalities might be more pronounced.

It is also possible that, given the communist past of the Czech Republic, socioeconomic residential segregation is, so far, less advanced in the Czech Republic than in Germany. In both cases, however, it can be hypothesized that the relatively weak effect of area-level characteristics on health behaviours in the Czech Republic may increase in the future with further economic growth and liberalization. As in other post-communistic countries this trend leads to an overall increase in living standards but simultaneously to widening inequalities between the social groups within the country [31].

But despite the variations which might reflect macro-social differences, the results are consistent at least for some of the risk factors. This is remarkable, because the dataset comprises nine towns in two different countries.

### Individual-level SES

The associations of socioeconomic status and risk factors were lower for area level SES than for individual level SES, which corresponds to previous findings [18]. It can be concluded that individual social background has a larger impact on lifestyle than the social environment. Especially education is a crucial factor for adopting or avoiding risk behaviour. But nonetheless our results show that area level SES seems to be an independent variable in relation to cardiovascular risk. According to these findings, interventions to reduce social inequalities should be more successful at individual level, but the highest impact can be expected for those measures which combine both individual (e.g. information campaigns about the benefit of physical activity) and structural prevention (e.g. improvement of local sport facilities) in neighbourhoods.

### Methodological considerations

When interpreting the results we have to be cautious because several aspects of the study design can influence them. First, the comparability of the analyses for Germany and the Czech Republic is restricted by the different median size of the administrative districts on which the neighbourhood level indicators rely. In Germany the units were approximately three times as large as in the Czech Republic. It has to be noted that larger units can be associated with a higher degree of misclassification of individuals with respect to their actual social environment. This effect can result in an underestimation of strength of positive associations [27]. However, the effects of area-level SES characteristics were more pronounced in Germany. This may suggest that the Czech-German differences are even larger.

Second, individual level socioeconomic status was measured by one indicator, because only education was assessed in a comparable way in HAPIEE and HNR. This is a limitation, because there could be a lack of consistency in individual and area level measures in terms of the underlying pathways to unequal health. Unemployment and overcrowding are primarily related to material circumstances, while education reflects social and cultural capital. The impact of this inconsistency on our results is probably limited, however, because participants' employment status, including unemployment, was included in the regression models. In addition a sensitivity analysis in the German sample, which contained a personal income variable, has shown that while adjustment for individual income and education reduced the odds ratios for the area-level variables, they remained significantly elevated.

Third, the response variable physical inactivity covers only one aspect of activities as it relies only on sports. Other aspects like non-sportive physical activities (e.g. working in the garden) or the daily walking distance to work or shopping are not included. As the importance of the neighbourhood environment may differ for these dimensions, further analysis with specific elements of physical activity are necessary [50].

Finally, as this study is cross-sectional, the uncertainty about the causality of the associations is higher than in a longitudinal design. For example, self selection of people with unhealthy lifestyles into low SES areas is possible, e.g. because of downward mobility. Although this is unlikely, we tried to minimize reverse causation by excluding all participants with a history of manifest myocardial infarction.

The limitations are balanced by strengths of this investigation. Our study comprises datasets from two countries and from nine cities with highly comparable measures of individual- and neighbourhood-level characteristics. Apart from the higher number of observations, the cross-national and cross-city design allows an analysis of differences between units and to assess consistency of associations in different settings. Both studies are sufficiently large and both were conducted to a high standard; this ensures a internal and external validity.

## Conclusion

Neighbourhood-level socioeconomic status was associated with some of the cardiovascular risk factors independent of individual social position. A strong and consistent association was found for smoking. Community based interventions could be seen as an appropriate instrument to reduce smoking prevalence and thus health inequalities, between urban neighbourhoods. Nonetheless, individual inequalities need to be taken into account when designing interventions, because they are strongly related to all types of risk behaviour under study.

## Competing interests

The author(s) declare that they have no competing interests.

## Authors' contributions

ND coordinated the neighbourhood level investigation for this paper, conducted data analyses and drafted the manuscript. HP, MB and NW participated in the coordination of this investigation, the data acquisition of the HNR and the HAPIEE Study, conducted analyses and helped drafting the manuscript. PEV conducted the multilevel analyses and contributed to draft the manuscript. AP, RK, SW, SM, SM and AS participated in data acquisition of the HNR and the HAPIEE Study and revised the manuscript. RE, KHJ and JS contributed to the acquisition of funding and revised the manuscript. All authors read and approved the final manuscript.

## Appendix (Advisory board and criteria and endpoint committee of the Heinz Nixdorf Recall Study)

Advisory board: T. Meinertz, Hamburg (Chair); M. Blettner, Mainz; C. Bode, Freiburg; PJ. de Feyter, Rotterdam, Netherlands; B. Güntert, Hall i.T., Austria; F. Gutzwiller, Switzerland; H. Heinen, Bonn; O. Hess, Bern; Switzerland; B. Klein, Essen; H. Löwel, Neuherberg; M. Reiser, Munich; G. Schmidt, Essen; M. Schwaiger, Munich; C. Steinmüller, Bonn; T. Theorell, Stockholm, Sweden; S.N. Willich, Berlin.

Criteria and endpoint committee: C. Bode, Freiburg (Chair); K. Berger, Münster; H.R. Figulla, Jena; C. Hamm, Bad Nauheim; P. Hanrath, Aachen; W. Köpcke, Münster; C. Weimar, Essen; A. Zeiher, Frankfurt.

## Pre-publication history

The pre-publication history for this paper can be accessed here:



## References

[B1] Marmot M, Wilkinson RG (2006). Social deterninants of health.

[B2] van Lenthe FJ, Siegrist J, Marmot M (2006). Aggregate deprivation and effects on health. Social inequalities in health.

[B3] Bosma H, van de Mheen HD, Borsboom GJ, Mackenbach JP (2001). Neighborhood socioeconomic status and all-cause mortality. Am J Epidemiol.

[B4] Marinacci C, Spadea T, Biggeri A, Demaria M, Caiazzo A, Costa G (2004). The role of individual and contextual socioeconomic circumstances on mortality: analysis of time variations in a city of north west Italy. J Epidemiol Community Health.

[B5] Martikainen P, Kauppinen TM, Valkonen T (2003). Effects of the characteristics of neighbourhoods and the characteristics of people on cause specific mortality: a register based follow up study of 252,000 men. J Epidemiol Community Health.

[B6] Osler M, Prescott E (2003). Educational level as a contextual and proximate determinant of all cause mortality in Danish adults. J Epidemiol Community Health.

[B7] Sloggett A, Joshi H (1994). Higher mortality in deprived areas: community or personal disadvantage?. BMJ.

[B8] Borrell LN, Diez Roux AV, Rose K, Catellier D, Clark BL, Atherosclerosis Risk in Communities Study (2004). Neighbourhood characteristics and mortality in the Atherosclerosis Risk in Communities Study. Int J Epidemiol.

[B9] Diez Roux AV, Borrell LN, Haan M, Jackson SA, Schultz R (2004). Neighbourhood environments and mortality in an elderly cohort: results from the cardiovascular health study. J Epidemiol Community Health.

[B10] Engstrom G, Goransson M, Hansen O, Hedblad B, Tyden P, Todt T, Janzon L (2000). Trends in long-term survival after myocardial infarction: less favourable patterns for patients from deprived areas. J Intern Med.

[B11] Macintyre K, Stewart S, Chalmer J, Pell J, Finlayson A, Boyd J, Redpath A, McMurray J, Capewell S (2001). Relation between socioeconomic deprivation and death from a first myocardial infarction in Scotland: population based analysis. BMJ.

[B12] Diez Roux AV, Merkin SS, Arnett D, Chambless L, Massing M, Nieto FJ, Sorlie P, Szklo M, Tyroler HA, Watson RL (2001). Neighborhood of residence and incidence of coronary heart disease. N Engl J Med.

[B13] Stjärne MK, Fritzell J, De Leon AP, Hallqvist J, SHEEP Study Group (2006). Neighborhood socioeconomic context, individual income and myocardial infarction. Epidemiology.

[B14] Chaix B, Rosvall M, Merlo J (2007). Recent increase of neighborhood socioeconomic effects on ischemic heart disease mortality: a multilevel survival analysis of two large Swedish cohorts. Am J Epidemiol.

[B15] Winkleby M, Sundquist K, Cubbin C (2007). Inequities in CHD incidence and case fatality by neighborhood deprivation. Am J of Prev Med.

[B16] Reijneveld SA (2002). Neighbourhood socioeconomic context and self reported health and smoking: a secondary analysis of data on seven cities. J Epidemiol Community Health.

[B17] Stafford M, Martikainen P, Lahelma E, Marmot M (2004). Neighbourhoods and self rated health: a comparison of public sector employees in London and Helsinki. J Epidemiol Community Health.

[B18] Pickett KE, Pearl M (2001). Multilevel analyses of neighbourhood socioeconomic context and health outcomes: a critical review. J Epidemiol Community Health.

[B19] Chaix B, Guilbert P, Chauvin P (2004). A multilevel analysis of tobacco use and tobacco consumption levels in France. Are there any combination risk groups?. Eur J Public Health.

[B20] Duncan C, Jones K, Moon G (1999). Smoking and deprivation: are there neighbourhood effects?. Soc Sci Med.

[B21] Kleinschmidt I, Hills M, Elliott P (1995). Smoking behaviour can be predicted by neighbourhood deprivation measures. J Epidemiol Community Health.

[B22] Reijneveld SA (1998). The impact of individual and area characteristics on urban socioeconomic differences in health and smoking. Int J Epidemiol.

[B23] Cubbin C, Sundquist K, Ahlen H, Johansson SE, Winkleby MA, Sundquist J (2006). Neighborhood deprivation and cardiovascular disease risk factors: Protective and harmful effects. Scand J Public Health.

[B24] Karvonen S, Rimpela A (1996). Socio-regional context as a determinant of adolescents' health behaviour in Finland. Soc Sci Med.

[B25] Cubbin C, Hadden WC, Winkleby MA (2001). Neighborhood context and cardiovascular disease risk factors: the contribution of material deprivation. Ethnicity & disease.

[B26] Sundquist J, Malmstrom M, Johansson SE (1999). Cardiovascular risk factors and the neighbourhood environment: a multilevel analysis. Int J Epidemiol.

[B27] Davey Smith G, Hart C, Watt G, Hole D, Hawthorne V (1998). Individual social class, area-based deprivation, cardiovascular disease risk factors, and mortality: the Renfrew and Paisley study. J Epidemiol Community Health.

[B28] van Lenthe FJ, Borrell LN, Costa G, Diez Roux AV, Kauppinen TM, Marinacci C, Martikainen P, Regidor E, Stafford M, Valkonen T (2005). Neighbourhood unemployment and all cause mortality: a comparison of six countries. J Epidemiol Community Health.

[B29] Bobak M, Pikhart H, Rose R, Hertzman C, Marmot M (2000). Socioeconomic factors, material inequalities, and perceived control in self-rated health: cross-sectional data from seven post-communist countries. Soc Sci Med.

[B30] Cornia AG, Paniccia R (2001). The Mortality Crisis in Transitional Economies.

[B31] McKeehan IV (2000). A mulitlevel city health profile of Moscow. Soc Sci Med.

[B32] Stang A, Moebus S, Dragano N, Beck E, Möhlenkamp S, Schmermund A, Siegrist J, Erbel R, Jöckel K (2006). Baseline recruitment and analyses of nonresponse of the Heinz Nixdorf Recall Study: Identifiability of phone numbers as the major determinant of response. Eur J Epidemiol.

[B33] Peasey A, Bobak M, Kubinova R, Malyutina S, Pajak A, Tamosiuna A, Pikhart H, Nicholson A, Marmot M (2006). Determinants of cardiovascular disease and other non-communicable diseases in Central and Eastern Europe: Rationale and design of the HAPIEE study. BMC Public Health.

[B34] Schmermund A, Möhlenkamp S, Stang A, Grönemeyer D, Seibel R, Hirche H, Mann K, Siffert W, Lauterbach K, Siegrist J, Jöckel KH, Erbel R (2002). Assessment of clinically silent atherosclerotic disease and established and novel risk factors for predicting myocardial infarctin and cardiac death in healthy middle-aged subjects: Rationale and design of the Heinz Nixdorf Recall Study. Am Heart J.

[B35] Pikhart H, Bobak M, Pajak A, Malyutina S, Kubinova R, Topor R, Sebakova H, Nikitin Y, Marmot M (2004). Psychosocial factors at work and depression in three countries of Central and Eastern Europe. Soc Sci Med.

[B36] Stang A, Moebus S, Möhlenkamp S, Dragano N, Schmermund A, Beck EM, Siegrist J, Erbel R, Jöckel KH, on behalf of the Heinz Nixdorf Recall Study Investigative Group (2006). Algorithms for converting Hawksley random zero to automated oscillometric blood pressure values and vice versa. Am J Epidemiol.

[B37] UNESCO (1997). International Standard Classification of Education ISCED 1997.

[B38] Berkman L, Glass T, Berkman L, Kawachi I (2000). Social integration, social networks, social support, and health. Social Epidemiology.

[B39] Berkman LF, Clark C, Kawachi I, Berkman LF (2003). Neighborhoods and networks: the construction of safe places and bridges. Neighborhoods and health.

[B40] Morris R, Carstairs V (1991). Which deprivation? A comparison of selected deprivation indexes. J Public Health Med.

[B41] Marsella AJ (1998). Urbanization, mental health, and social deviancy. A review of issues and research. Am Psychol.

[B42] Chaix B, Rosvall M, Lynch J, Merlo J (2006). Disentangling contextual effects on cause-specific mortality in a longitudinal 23-year follow-up study: impact of population density or socioeconomic environment?. Int J Epidemiol.

[B43] Breslow NE, Clayton DG (1993). Approximate inference in generalized linear mixed models. J Am Stat Assoc.

[B44] Chuang YC, Cubbin C, Ahn D, Winkleby MA (2005). Effects of neighbourhood socioeconomic status and convenience store concentration on individual level smoking. J Epidemiol Community Health.

[B45] van Lenthe FJ, Mackenbach JP (2006). Neighbourhood and individual socioeconomic inequalities in smoking: the role of physical neighbourhood stressors. J Epidemiol Community Health.

[B46] Curry SJ, Wagner EH, Cheadle A, Diehr P, Koepsell T, Psaty B, McBride C (1993). Assessment of community-level influences on individuals' attitudes about cigarette smoking, alcohol use, and consumption of dietary fat. Am J Prev Med.

[B47] Siegrist J, Marmot M (2006). Social inequalities in health New evidence and policy implications.

[B48] Sing-Manoux A, Marmot M (2005). Role of socialization in explaining social inequalities in health. Soc Sci Med.

[B49] Chaix B, Chauvin P (2003). Tobacco and alcohol consumption, sedentary lifestyle and overweightness in France: A multilevel analysis of individual and area-level determinants. Eur J Epidemiol.

[B50] van Lenthe FJ, Brug J, Mackenbach JP (2005). Neighbourhood inequalities in physical inactivity: the role of neighbourhood attractiveness, proximity to local facilities and safety in the Netherlands. Soc Sci Med.

